# A Platform to Develop and Apply Digital Methods for Empirical Bioethics Research: Mixed Methods Design and Development Study

**DOI:** 10.2196/28558

**Published:** 2022-05-05

**Authors:** Manuel Schneider

**Affiliations:** 1 Health Ethics and Policy Lab Department of Health Sciences and Technology ETH Zurich Zurich Switzerland

**Keywords:** digital bioethics, digital humanities, digital methods, computational methods, empirical bioethics, research platform, digital health, bioethics, digital platform

## Abstract

**Background:**

The rise of digital methods and computational tools has opened up the possibility of collecting and analyzing data from novel sources, such as discussions on social media. At the same time, these methods and tools introduce a dependence on technology, often resulting in a need for technical skills and expertise. Researchers from various disciplines engage in empirical bioethics research, and software development and similar skills are not usually part of their background. Therefore, researchers often depend on technical experts to develop and apply digital methods, which can create a bottleneck and hinder the broad use of digital methods in empirical bioethics research.

**Objective:**

This study aimed to develop a research platform that would offer researchers the means to better leverage implemented digital methods, and that would simplify the process of developing new methods.

**Methods:**

This study used a mixed methods approach to design and develop a research platform prototype. I combined established methods from user-centered design, rapid prototyping, and agile software development to iteratively develop the platform prototype. In collaboration with two other researchers, I tested and extended the platform prototype in situ by carrying out a study using the prototype.

**Results:**

The resulting research platform prototype provides three digital methods, which are composed of functional components. This modular concept allows researchers to use existing methods for their own experiments and combine implemented components into new methods.

**Conclusions:**

The platform prototype illustrates the potential of the modular concept and empowers researchers without advanced technical skills to carry out experiments using digital methods and develop new methods. However, more work is needed to bring the prototype to a production-ready state.

## Introduction

Empirical bioethics is an interdisciplinary research field attracting researchers with various backgrounds [1]. Software development skills and similar know-how are often not part of their expertise. However, inquiries in the field of empirical bioethics can rely heavily on computational tools (the textual analysis of millions of tweets, for example). From the 1990s, social scientists have recognized the internet as a valuable research subject and data source, and have adapted their methods and tools to novel digital phenomena [2,3]. This recognition has resulted in novel digital methods*,* which Snee et al [4] define as “the use of online and digital technologies to collect and analyse research data.” Novel disciplines emerged, such as computational social science, which leverages computational capabilities to collect and analyze big data, to study social behavior [5].

The development of these methods has spawned a variety of digital tools. One example is the network visualization software Gephi [6]. Gephi imports different data formats and provides functionality to researchers through a graphical user interface. Researchers can use this software to compute basic network statistics, such as network density and average shortest path length. Network visualization features build the core of Gephi, allowing researchers to explore and manipulate large networks. Gephi is open source, and can integrate plugins, enabling software developers to extend the functionality.

Alongside the maturation of digital methods, advances in computer science, especially machine learning, have resulted in an abundance of software libraries. One example is Hugging Face, a Python library that provides state-of-the-art natural language processing (NLP) resources [7]. Hugging Face allows researchers to program their own NLP pipelines; for example, to enable analysis of the sentiment of tweets. In contrast to Gephi, Hugging Face is a collection of resources used by developers to write software programs. It does not offer a graphical user interface or out-of-the-box workflows for researchers without programming experience. One advantage of such a library is that developers have a great deal of control over how to use the resources, which can also be easily combined with resources from other libraries.

In light of these new developments and tools, and in collaboration with other researchers, I conducted several empirical bioethics experiments using digital methods, hereinafter referred to as “digital bioethics.” My collaborators and I encountered two major issues over the course of these experiments: (1) the need for technical expertise to set up tools and adapt them to each experiment, and (2) the need for technical expertise to develop new methods. In my observation, finding ad hoc expertise can delay projects and make it more difficult for researchers to conduct digital bioethics experiments. If researchers could easily access tools and seamlessly integrate them into their research projects without the assistance of software developers, they might be more inclined to practice digital bioethics.

Researchers would still rely on software development skills to develop new tools when fundamentally new functionality is required. However, existing tools might be repurposed, modified, and recombined, if they were built with that objective in mind. Therefore, I aimed to develop a research platform which addresses the two identified issues. In the following, I describe the development process, the resulting research platform prototype, and the learnings from this process.

## Methods

### Overview

**Figure 1:**
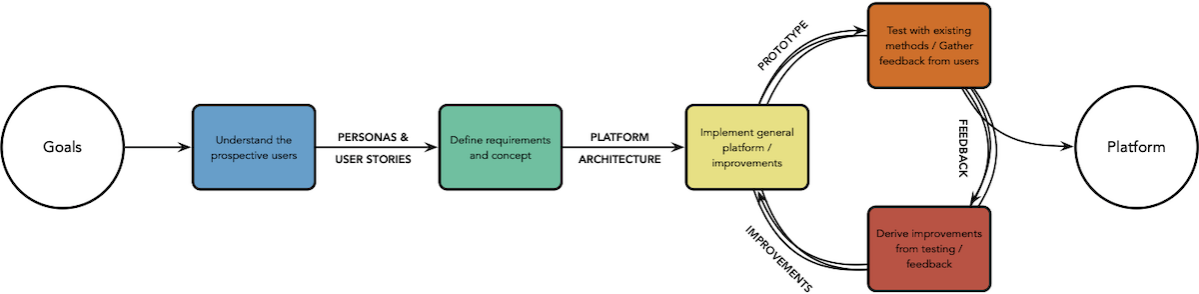
Overview of the methods. The circles represent the start and end points of the development process, and the colored boxes represent the work steps. The three boxes on the right form a prototyping cycle, which I carried out multiple times during the development process. The text in between the steps describes the main inputs and outputs of a step.

Using a combination of user-centered design [8], rapid prototyping [9], and agile software development [10], I developed a platform prototype that addresses issues (1) and (2) introduced in the *Introduction*. Rather than regarding these issues only as challenges, I translated them into goals, which express an ideal scenario: (A) researchers can easily configure and employ methods provided by the platform for new experiments, and (B) researchers can modify methods and develop new methods, by recombining components of already implemented digital methods. These high-level goals guided the development process, which I describe in the following paragraphs (see Figure 1 for an overview of the approach).

### Understanding the Prospective Users

The first step of the development process was to create *personas*; that is, abstract individuals who represent typical target users [11,12]. In total, I created four personas (two for each goal [A] and [B]). I defined their attributes such as name, age, gender, and professional background, as well as their personal goals, challenges, and motivations with respect to digital bioethics. I based the personas on my own experience, as well as anecdotal data from colleagues and the empirical bioethics literature [1,13,14], which is common practice when no empirical data are available. I then derived so-called *epics,* that is high-level narratives of what each persona as a user would want to do on the research platform, and why [15]. The epics incorporated the platform goals (A) and (B) from the perspective of the persona. Finally, I broke down the epics into specific tasks a user might want to accomplish, together with the user’s corresponding motivation [8]. These *user stories* took the form “as a <user> I want to <action> so that <value>,” commonly referred to as the role-feature-reason format [16]. The user stories formed the initial functional requirements for the platform and described the user-centered features (see Multimedia Appendices 1 and 2 for the personas, epics, and user stories).

### Designing the Platform Concept

While the user stories described the intended functionality of the platform, they also had nonfunctional implications for the platform design. For example, if two researchers were working on a project at the same time, the actions of one researcher should not unintentionally override data resulting from actions of the other researcher. I also defined nonfunctional requirements from my past experience with digital bioethics. As an example, training a machine learning model requires significant computational power and is time-consuming. I therefore defined requirements about the performance of the platform and its ability to run such a time-consuming process without blocking other processes. I then designed the high-level platform architecture based on these requirements, using a micro service and micro frontend approach [17-19].

### Implementing the Base Prototype

In the final phase of the development process, I used rapid prototyping [9,20] to implement a first functional prototype, and then to iteratively improve the prototype through evolutionary prototyping (ie, continuously improving the same prototype). Initially, I implemented the overall platform without incorporating any specific digital method, focusing on general platform functionality such as data handling and the graphical user interface (GUI). Next, I implemented two digital methods: one from a study examining the web-based data sharing policy landscape [21], and another from a study analyzing themes in tweets about CRISPR [22]. To adhere to goal (B) and the corresponding user stories, I did not implement the methods as one monolithic process, but rather split into functional components. For example, I implemented a data filtering step as an individual component, and not as part of a multi-stage data processing pipeline. The implementation of these methods allowed me to test the platform’s conceptual choices and added functionality to the platform prototype at the same time.

### Testing and Improving the Prototype in a Real-life Scenario

To test the prototype in a real-life scenario, I collaborated with two researchers (Julia Amann, Joanna Sleigh) from the same lab to investigate visual risk communication about COVID-19 on Twitter [23]. I completed multiple prototyping iterations with one researcher (JS) to implement functionality that was necessary for the research, but not yet implemented in the platform. Over the course of the study, I continuously gathered feedback from the researchers about their user experience, defined improvements based on this feedback, and implemented the improvements, forming multiple prototyping cycles (see Figure 1). The researchers also requested additional features based on their experiences with the platform. I implemented, tested, and improved the requested features in-situ; the researchers used the new features over the course of the study and provided direct feedback, until the features fit their needs precisely. At the conclusion of the visual communication study, the platform prototype contained all components of the study methods.

## Results

### Overview

The main result of this study is the research platform prototype. It is important to note that the goal of this study was to develop a platform that affords more flexibility to a researcher working with digital methods. Therefore, the main focus of this section is to describe how the platform provides this flexibility, and not how a specific digital method is implemented. In the following, I report the major technical and functional design choices, and describe the main features of the platform (see Multimedia Appendix 3 for screenshots of the platform prototype and descriptions thereof).

### Platform Architecture

The platform is implemented as a client-server model, allowing multiple users to work on the same project with all data centrally stored. This model eliminates the peer-to-peer sharing of data sets but imposes security features on the platform. Client-server communication implements the standard HTTPS protocol with Transport Layer Security encryption. Users are authenticated through the Authentication and Authorization Infrastructure [24] provided by most Swiss universities, and can share projects with other authenticated users.

**Figure 2:**
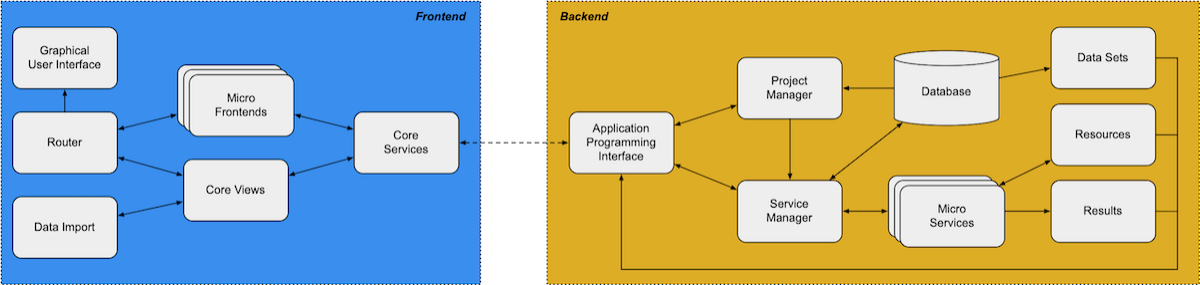
High-level overview of the platform architecture. The platform is separated into the frontend, with which the user interacts (left), and the backend, which is concerned with data storage and processing (right). The two communicate through HTTPS (dashed line). The micro frontends and micro services provide the functionality of the individual components, which together represent the digital methods. The arrows indicate the direction of communication flow between the elements of the platform.

The frontend was realized as a web application using the web application framework Angular [25] to be independent of the user’s operating system, and supports browsers compliant with the World Wide Web Consortium’s web standards [26]. The backend was built with the Python framework Flask [27] and employs the Python library pandas [28] for data management and core data operations, such as extending data sets with data sent from the frontend and providing dataset previews to the frontend. Packages are managed through a Python virtual environment. An overview of the platform architecture is shown in Figure 2.

The GUI defines the web application’s layout and style, such as colors and fonts. A router enables the user to navigate to the different core views that provide the general functionality of the platform. When prompted by the user, the router provides the custom digital method specific views (micro frontends) to the GUI. Two core frontend services facilitate communication with the backend, and are both available to the core view and micro frontends.

The backend’s application programming interface (API) allows the frontend to interact with the backend functionality. Through the API, the frontend can load and store information about research projects, handled by the project manager. The actions carried out and the data processed when running a project are coordinated by the service manager, which starts and stops micro services, and reads and writes data. The micro services can store resources (such as images) and results (such as tables or figures resulting from the analysis) as files which can be requested from the frontend through the API. To download a data set from the web application, a user can request the specific data set from the database through the API.

The micro services and micro frontends form the heart of the platform. When a digital method is implemented on the platform, the method is broken down into smaller units, each fulfilling one specific function. These units are implemented as either an interactive *task* or a fully automated *process*, which can be chained together in a *pipeline*. The tasks and processes both process data, receive different kinds of input, and produce results; for example, a chart illustrating the outcome of a statistical analysis carried out as part of the process. An abstract micro service class and an abstract micro frontend class provide all of the technical functionality necessary to seamlessly integrate new tasks and processes implemented by a developer.

A task consists of a micro service, which carries out operations requested by the user, and a micro frontend, which provides the user with a custom user interface for the task. This interface allows the user to trigger operations of the micro service and to exchange data with it. The user starts a task through the frontend, performs actions through the task-specific interface, and stops the task once it is completed, all in a synchronous way. In contrast, a process is started by the user, and runs asynchronously until completion. In this context, *asynchronous* means that the user can perform other actions while the process is running, and can even close the web application. This mechanism is intended for time-intensive operations that can continue for multiple hours or days, such as analyzing a large amount of data or training a machine learning model.

All tasks and processes have a specification file, which defines what input data they take, what data they output, what results they produce, and what parameters the user can configure. Thanks to this shared core concept, a user can easily connect and configure tasks and processes. I will explain how this is done, together with the other main features of the platform, in the next subsection.

### Main Features of the Web Application

**Figure 3:**
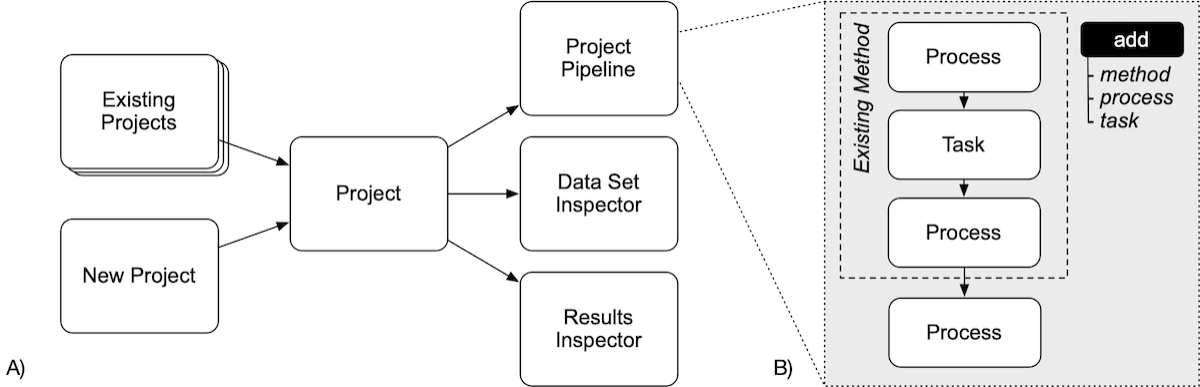
Main features of the web application. Experiments are organized into projects which offer three main functionalities: the project pipeline where the experiment is run, the data inspector that provides insight into the data sets of the experiments, and the results inspector displaying the results produced by the components (A). The user can populate the pipeline with existing methods, tasks, and processes by adding them from collections (B).

On the starting page of the platform, a user can choose to create a new project by specifying a project name and description, or to open an existing project. A project consists of the project pipeline, data set inspector, and results inspector (see Figure 3A).

**Figure 4:**
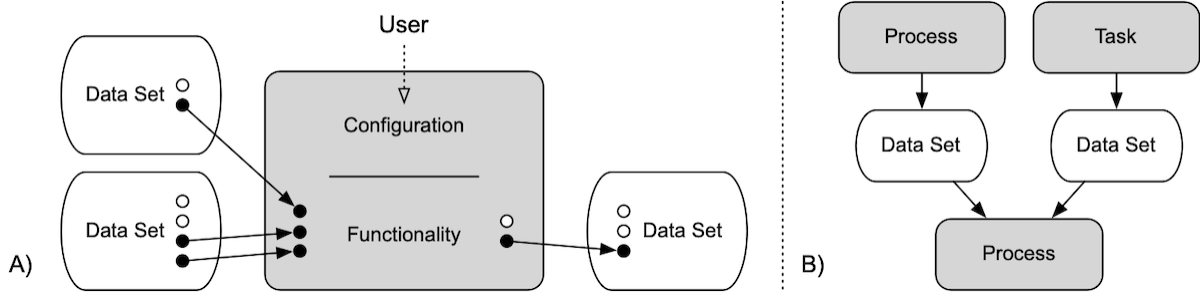
Integration of the components into the pipeline. Components (processes and tasks) are configured by the user. Besides functionality-specific parameters, the user also specifies which data sets provide the input data to each component, and what output data is written to which data set (A). The components’ relations to the input and output data sets define the execution sequences, and are reflected in the pipeline (B).

The project pipeline is the control center for the project. It displays all processes and tasks carried out during the course of the project, and lets the user start them individually (see Figure 3B). When a new project is created, the user can choose an existing digital method from a collection of implemented methods. The pipeline is then populated with all components (ie, tasks and processes) of that method. The user can configure each task and process to match the context of the current project. The user can also specify which datasets provide the input data for each component, and whether the output data forms a new data set or is appended to an existing one (see Figure 4A).

Alternatively, the user can develop a new method or adapt an existing method by adding individual processes and tasks to the pipeline. These processes and tasks are then connected to components through their input and output data sets (see Figure 4B). If a new method is created or an existing method changed, the user can export the pipeline to the method collection and provide a rationale for the methodological choices in a text field. The user can ultimately run a project by starting processes and tasks in the pipeline through a click on the respective button.

Two distinct features help a researcher to keep track of the ongoing project. The data set inspector shows all existing data sets for a project, and a preview of the data. This enables researcher oversight, to see that the processes and tasks are functioning as intended. The data set inspector also allows the user to download full datasets for further inspection. Similarly, the results inspector shows all results (such as charts) produced as part of the tasks and processes. The results can also be downloaded from the results inspector.

The last feature I discuss here is the knowledge base. A detailed understanding of how the methods and components work is of great importance for the development and application of digital methods. To address this challenge, each component and method has a detailed description. The description is adapted automatically on the basis of the user’s configuration of the component, to reflect the actual pipeline as accurately as possible. These descriptions serve to educate users about available components and methods and help users assess their suitability for a new study design. The descriptions also allow users to verify that the composed pipeline does what they intended it to do and to accurately describe the method; for example, in a scientific publication about a study conducted on the platform.

### Visual Communication Study

**Figure 5:**
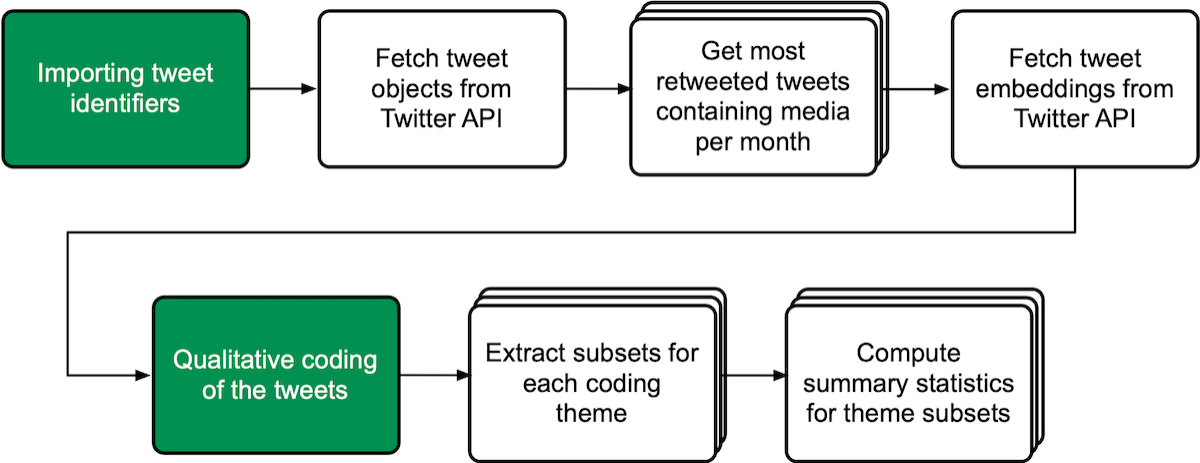
Overview of the visual communication study pipeline. The researchers carried out the tasks (in green) while the other elements represent the process components, which automatically carried out functions. Process components with the same or similar functionality are grouped for simplicity (overlapping elements). API: application programming interface.

The communication study [23] conducted on the platform prototype during the last phase of the development process exemplifies how researchers can use the platform for their research. Hence, I characterize the project pipeline of the study and describe the procedure the researchers followed on the platform (see Figure 5 for a schematic overview of the pipeline).

The communication study’s pipeline consisted of 2 tasks and 23 processes. Some of the process components performed the same function. For example, the statistical analysis at the end of the pipeline employed the same process component type multiple times. However, the components’ individual configurations resulted in distinct statistics describing different aspects of the data. The pipeline view (the control center of the project) allowed the researchers to start tasks and processes. When researchers started a task, the platform prompted a custom task interface, which allowed them to carry out each task’s specific work. Researchers ran the processes one after another by clicking on the respective button in the pipeline view. It was in this way that data was processed throughout the pipeline. Altogether, the tasks and components formed 7 functional steps (see Figure 5).

The researchers started their work on the platform by importing the tweet identifiers from a data set they obtained from Crowdbreaks [29], which collects tweets concerning various public health topics. They then ran a process component that fetched the entire tweet object (eg, tweet text, hashtags, and retweet count) for each tweet identifier from the Twitter API. The subsequent processes filtered the data set to include only tweets that contained visuals and selected the 500 most retweeted tweets per month.

The process that followed fetched tweet embeddings (formatted text snippets) from the Twitter API, which the subsequent task utilized to display the tweets within its custom interface. The task interface offered two views to carry out the qualitative coding, a form of qualitative content analysis [30], representing the researchers’ main activity during the study. One view presented the individual tweets and allowed the researchers to select items matching the tweets’ characteristics from a predefined codebook specified in the task’s configuration (see Multimedia Appendix 3 for a screenshot). A second view displayed a list of all the tweets providing a preview for each tweet, the initials of the coders that coded it, and whether or not it was included in the analysis.

Following the coding task, multiple process components extracted subsets from the coding data set, one subset for each of the 6 coding themes. Finally, 12 process components provided statistical results for all the themes, which created the basis for the researchers’ manual inspection and interpretation of the results. The statistical processes also produced basic figures from the statistical results to assist interpretation (see Multimedia Appendix 3 for a screenshot).

## Discussion

### Principal Findings

The study using the prototype to examine visual communication on Twitter [23] demonstrates the potential of the platform design. In accordance with the platform’s modular design, I implemented the study method as individual components. For example, the platform provides a component to carry out a qualitative coding of tweets. Now that the study components are available on the platform, the visual communication study pipeline can be used as a template for similar studies. As the functionality of the qualitative coding component is not limited to tweets, it can be used to analyze other content (for example news articles) as well.

The platform prototype offers greater flexibility to researchers without programming skills, when compared with Gephi or Hugging Face. Once a new digital method is implemented on the platform, a researcher can easily configure the method to fit a specific experiment, or adapt an existing method by removing and adding components. Further, a researcher can develop a new method by combining individual components of existing methods. If new functionality is needed, software developers can readily integrate a new component, thanks to the platform’s modular architecture.

Enabling researchers to function with greater independence from technical experts might seem counterintuitive for an interdisciplinary endeavor such as digital bioethics. I do not suggest excluding technical experts; my aim is rather to minimize purely implementation-related technical work.

The knowledge feature only emerged during the rapid prototyping phase and illustrates the importance of training and educating researchers new to digital methods. Such a research platform should not only be a tool to develop methods and carry out research projects, but also a means for researchers to acquire skills and expertise necessary for digital methods research.

### Limitations

The development of new components can still cause delay for research projects and needs resources from both researchers and software developers. However, the platform can reduce software development work, as researchers can configure and reuse components outside of the specific circumstances for which they were originally developed. Furthermore, if multiple researchers work with the platform and develop methods with this modular paradigm, the methodological flexibility increases, and established computational means can be combined in new ways. Although maintenance and extension of the platform must be performed by technical experts, the application of digital methods in digital bioethics research can scale.

The prototype illustrates the potential of the implemented concept. However, more work is required to obtain to a fully operational research platform that can support a broad community of researchers. Further, I could not test the platform exhaustively owing to the limited number and availability of researchers working with digital methods in bioethics. For the same reason, I could not carry out comprehensive user research during the design phase. In such cases, the use of fictional personas based on limited assumptions, as applied in the platform development, is common practice. In addition, I was able to validate my assumptions and improve the design choices during the rapid prototyping phase at the end.

### Conclusions

The platform prototype is a proof of concept, demonstrating how this approach might facilitate digital bioethics research by offering researchers easier access to digital tools and opportunities to expand their methods toolbox. Further research is needed to quantify this effect and to further improve the platform. In addition, substantial investment will be required to build, run, and maintain a production-ready open research platform, for which this prototype serves as a blueprint. While I built this platform to address issues encountered in digital bioethics research, it does support digital methods in general, which are not restricted to bioethics research. Digital methods find applications in various research fields and, therefore, this platform and its concept are useful beyond empirical bioethics.

## Appendix

Multimedia Appendix 1 Personas and Epics.

Multimedia Appendix 2 User Stories.

Multimedia Appendix 3 Screenshots of Platform User Interface.

## Abbreviations

API: application programming interface
GUI: graphical user interface
NLP: natural language processing
