# Supplementary material for: A Platform to Develop and Apply Digital Methods for Empirical Bioethics Research: Mixed Methods Design and Development Study
Source: JMIR Form Res. 2022 May 5;6(5):e28558. doi: 10.2196/28558 (PMC9121222; doi:10.2196/28558)
Supplement: Multimedia Appendix 1 [file formative_v6i5e28558_app1.pdf]

Multimedia Appendix 1 – Personas and Epics

Using Digital Methods – Goal (A)

Mary	Fred
**Age**: 28<br>**Gender**: Female<br>**Occupation**: PhD Student in Medical Ethics<br>**Background**: MSc in Medicine, received basic training of machine learning in medicine<br>**Goals:**<br>- study of ethical implications of public debate surrounding mental illnesses<br>- temporal and real-time analysis of conversations on social media<br>**Challenges:**<br>- managing a research project<br>- vast amount of textual data<br>- implementing end-to-end data processing pipeline<br>**Motivations:**<br>- access to big data source<br>- possibility to analyze individual statements<br>- possibility to analyze user networks<br>- fast data processing<br><br>**Epic:** On the platform, I want to use digital methods for social media analysis to collect tweets containing certain keywords and analyze the statements and connections between users.	**Age**: 47<br>**Gender**: Male<br>**Occupation**: Senior Researcher<br>**Background**: MA in International Law, PhD in Public Health, lecturer in qualitative research methods<br>**Goals:**<br>- examine the adoption of the sustainable development goals into national and international public health policies from 2015 until 2020<br>**Challenges:**<br>- no programming skills<br>- no structured data source<br>**Motivations:**<br>- data mining from web resources<br>- processing of large textual data set<br>- automated thematic probing<br><br>**Epic:** On the platform, I want to use digital methods to collect public health policies from the internet, extract content and probe the presence of known themes in the textual data.

Developing Digital Methods – Goal (B)

Peter	Emily
**Age**: 33<br>**Gender**: Male<br>**Occupation**: Post-Doctoral Researcher<br>**Background**: MA in Sociology, PhD in Global Health, expert in computational methods for big data analysis<br>**Goals:**<br>- investigate the use of social network analysis and topic modelling to understand the dissemination dynamics of health-related information on social media<br>**Challenges:**<br>- limited programming skills<br>- no out-of-the box solutions<br>- methodological experimentation<br>**Motivations:**<br>- huge dataset of posts worldwide<br>- exact timestamps of posts<br>- modelling and simulation of dissemination<br><br>**Epic:** On the platform, I want to experiment with existing computational tools to find new methods to study the dissemination of global health-related information.	**Age**: 58<br>**Gender**: Female<br>**Occupation**: Professor of Medical Ethics<br>**Background**: MA in Philosophy, PhD in Bioethics, lecturer in grounded theory<br>**Goals:**<br>- development of automated conceptual analysis combined with qualitative coding<br>**Challenges:**<br>- no programming skills<br>- coordination efforts with programmers<br>**Motivations:**<br>- adapt qualitative research methodology to big data sources<br>- investigate online phenomena like social media<br><br>**Epic:** On the platform, I want to combine computational and digital forms of qualitative research methods to automatically derive concepts from large corpora of documents.
