# Supplementary material for: A Platform to Develop and Apply Digital Methods for Empirical Bioethics Research: Mixed Methods Design and Development Study
Source: JMIR Form Res. 2022 May 5;6(5):e28558. doi: 10.2196/28558 (PMC9121222; doi:10.2196/28558)
Supplement: Multimedia Appendix 2 [file formative_v6i5e28558_app2.pdf]

Multimedia Appendix 2 – User Stories

Using Digital Methods – Goal (A)

The user stories incorporate the research the personas conduct. Duplicate user stories were not added twice.

as a <user>	*I want to <action>*	*so that <value>*
student	use existing project as a template	I know the right setup for the method
student	share a research project	I can involve my supervisor
student	specify the parameters for the data collection process	the data of interest is collected
student	start the data collection	the data is collected and stored
student	preview the collected data	I better understand the data at hand
student	see summary statistics of the data	I better understand the data at hand
student	run analysis scripts	I get results
student	view the results	I can interpret the results
researcher	start a new research project from a template	I can use an existing method
researcher	add other researchers to my project	I can collaborate
researcher	specify the data collection parameters	I get the right data
researcher	combine two existing methods	I can run the whole experiment in one project
researcher	examine the collected data	I can check that the correct data is collected
researcher	input existing data	It can be used in combination with collected data
researcher	export data	I can use other tools for tasks like visualization
researcher	export result products (figures, …)	I can use them for publications
researcher	download a project	I can archive it

Developing Digital Methods – Goal (B)

User stories that were already present in the previous table (using digital methods) were not added again.

as a <user>	*I want to <action>*	*so that <value>*
post-doc	use existing project as a template	I can modify the method
post-doc	add and remove elements to the method	I can test modified approaches
post-doc	run the method	I can examine the effect of the changes
post-doc	specify alternative data fields to use for an analytical step	I can see if the analysis can also be used for similar data types
professor	start a blank project	I can build the method from scratch
professor	add elements to the method	I can compose the method
professor	interact with the data	I can carry out qualitative tasks
professor	export data	I can examine steps of the method in detail
professor	link parts of the method	I don’t have to manually transfer data
