# Supplementary material for: A Platform to Develop and Apply Digital Methods for Empirical Bioethics Research: Mixed Methods Design and Development Study
Source: JMIR Form Res. 2022 May 5;6(5):e28558. doi: 10.2196/28558 (PMC9121222; doi:10.2196/28558)

Multimedia Appendix 3 – Screenshots of Platform User Interface

The screenshots are taken from the visual communication study project but with example data and example codebook.

Processing Pipeline

The processing pipeline allows the user to get an overview of the method and run the components.

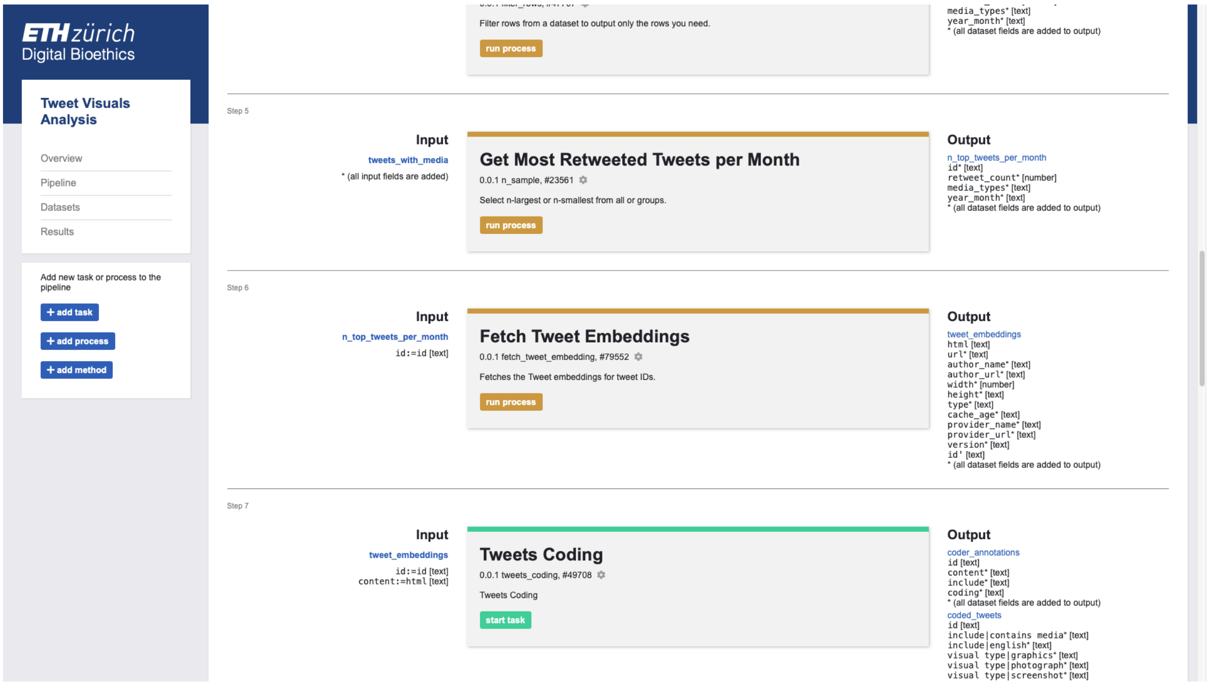

Data Inspector

The data inspector lists the datasets and gives a preview of the data. Hyperlinks and other datatypes are automatically detected and shown accordingly.

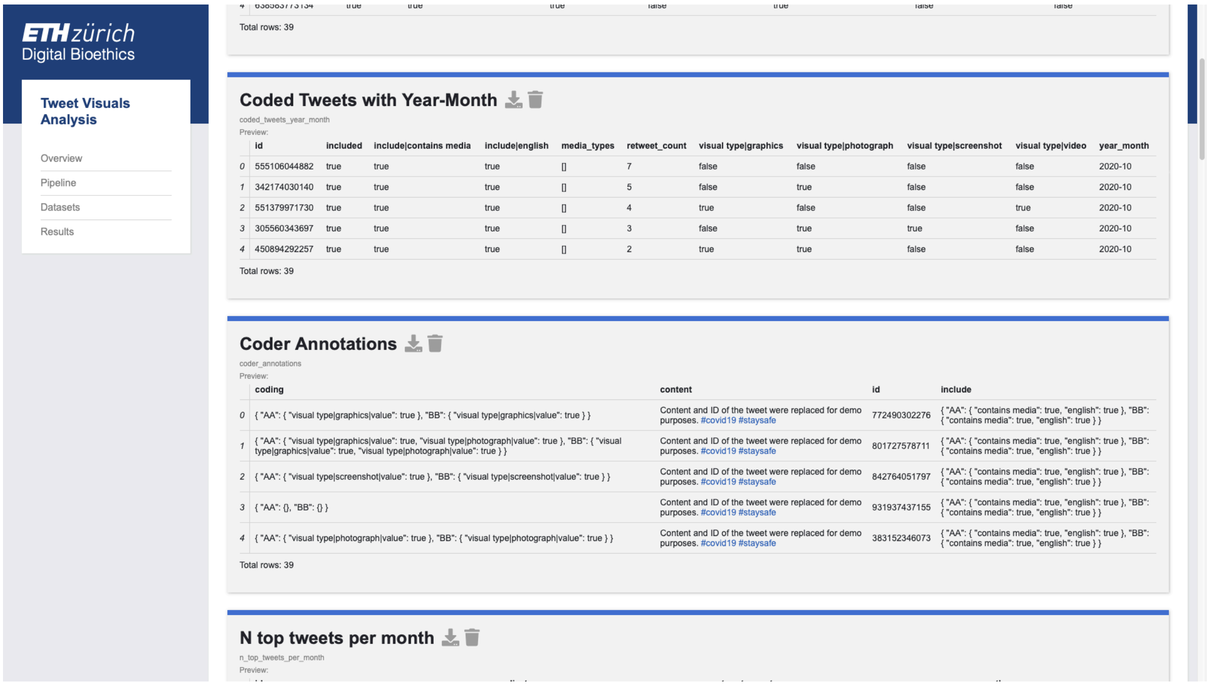

Component Configuration

The user can specify the configuration of tasks and processes, for example which fields of a dataset are used as the input to the component.

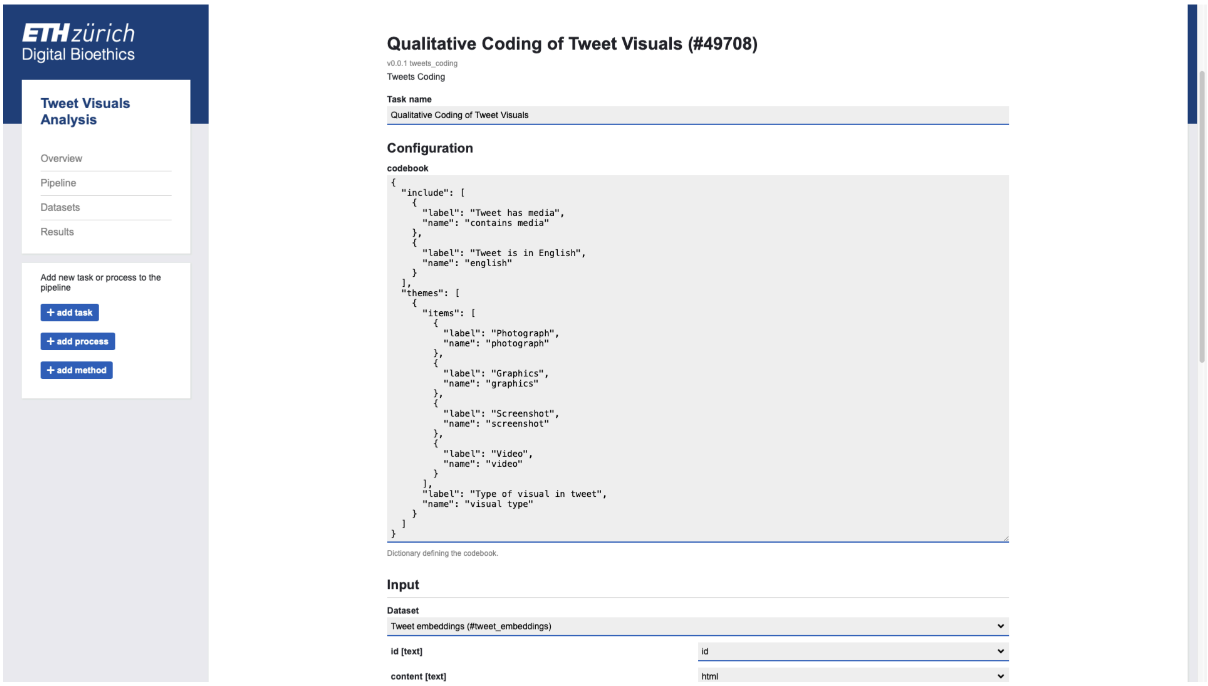

Task Interface

This example shows the custom interface for the qualitative coding of tweets.

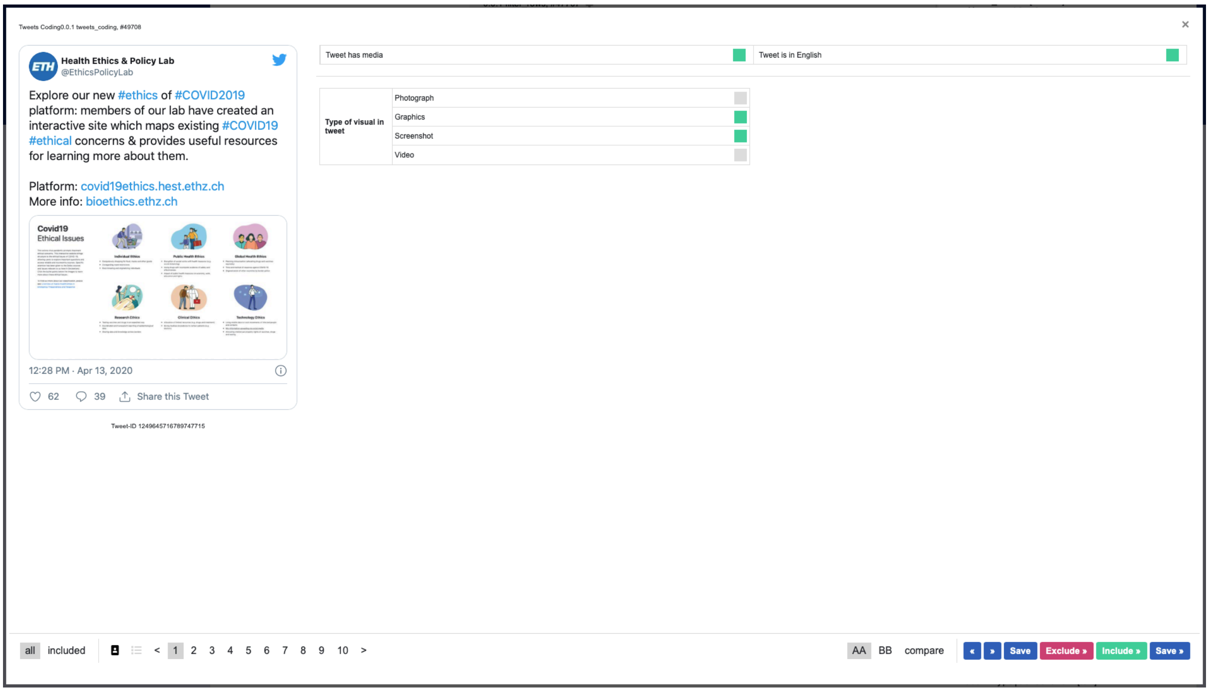

Results Inspector

The results inspector displays the outputs produced by processes and tasks and allows the user to inspect and download them.

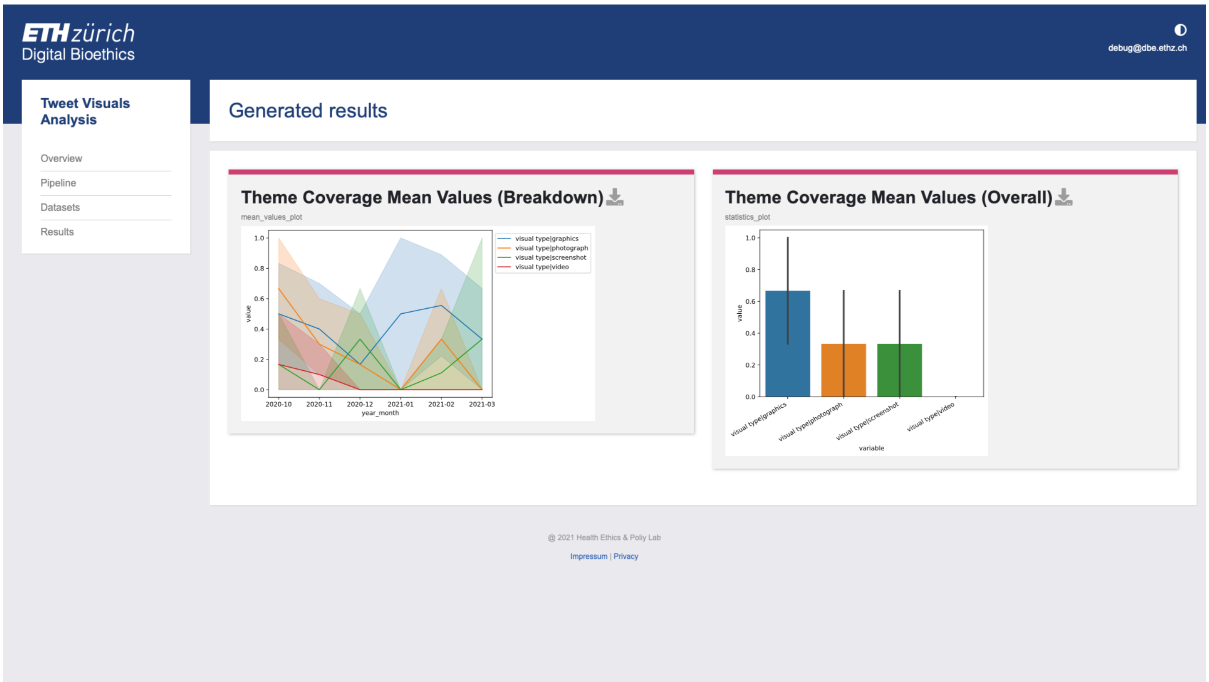

Supplement: Multimedia Appendix 3 [file formative_v6i5e28558_app3.pdf]
